# Pinin interacts with C-terminal binding proteins for RNA alternative splicing and epithelial cell identity of human ovarian cancer cells

**DOI:** 10.18632/oncotarget.7242

**Published:** 2016-02-08

**Authors:** Yanli Zhang, Jamie Sui-Lam Kwok, Pui-Wah Choi, Minghua Liu, Junzheng Yang, Margit Singh, Shu-Kay Ng, William R. Welch, Michael G. Muto, Stephen KW Tsui, Stephen P. Sugrue, Ross S. Berkowitz, Shu-Wing Ng

**Affiliations:** ^1^ Laboratory of Gynecologic Oncology, Division of Gynecologic Oncology, Department of Obstetrics, Gynecology and Reproductive Biology, Harvard Medical School, Boston, MA, USA; ^2^ School of Biomedical Sciences, The Chinese University of Hong Kong, Hong Kong; ^3^ Department of Anatomy and Cell Biology, University of Florida College of Medicine, Gainesville, FL, USA; ^4^ School of Medicine and Menzies Health Institute Queensland, Griffith University, Meadowbrook, Australia; ^5^ Department of Pathology, Brigham and Women's Hospital, Harvard Medical School, Boston, MA, USA

**Keywords:** ovarian cancer, tumorigenesis, RNA metabolism, cell adhesion, RNA sequencing

## Abstract

Unlike many other human solid tumors, ovarian tumors express many epithelial markers at a high level for cell growth and local invasion. The phosphoprotein Pinin plays a key role in epithelial cell identity. We showed that clinical ovarian tumors and ovarian cancer cell lines express a high level of Pinin when compared with normal ovarian tissues and immortalized normal ovarian surface epithelial cell lines. Pinin co-localized and physically interacted with transcriptional corepressor C-terminal binding proteins, CtBP1 and CtBP2, in the nuclei of cancer cells. Knockdown of Pinin in ovarian cancer cells resulted in specific reduction of CtBP1 protein expression, cell adhesion, anchorage-independent growth, and increased drug sensitivity. Whole transcriptomic comparison of next-generation RNA sequencing data between control ovarian cancer cell lines and cancer cell lines with respective knockdown of Pinin, CtBP1, and CtBP2 expression also showed reduced expression of CtBP1 mRNA in the Pinin knockdown cell lines. The Pinin knockdown cell lines shared significant overlap of differentially expressed genes and RNA splicing aberrations with CtBP1 knockdown and in a lesser degree with CtBP2 knockdown cancer cells. Hence, Pinin and CtBP are oncotargets that closely interact with each other to regulate transcription and pre-mRNA alternative splicing and promote cell adhesion and other epithelial characteristics of ovarian cancer cells.

## INTRODUCTION

Ovarian cancer has the highest mortality rate of all gynecologic malignancies and is the fifth leading cause of cancer death in females in the United States [1]. The majority of patients with serous epithelial cancer, the most common epithelial ovarian malignancy, were usually diagnosed at an advanced stage and had a 5-year survival rate of less than 25% and a 10-year survival rate approaching zero [1]. The high death rate is not only due to the advanced stage of disease at diagnosis but also to the lack of disease-specific and effective therapy. Therefore, it is of paramount importance to understand the underlining mechanisms by which ovarian pathogenesis and tumor progression are regulated and to identify clinical targets for therapeutic development.

Characterization of ovarian tumors and cancer cell lines has shown that they are highly proliferative and are more epithelial-like than normal ovarian surface epithelia and the derived cell lines, which are mesothelial cells in nature [2–5]. Benign metaplastic ovarian cysts and the associated ovarian tumor cells [2, 3] express high levels of E-cadherin [6] and show suppression of tumor growth factor-beta (TGF-β) pathway [7]. Ectopic expression of E-cadherin caused mesenchymal-epithelial transition (MET) in ovarian surface epithelial cells and tumor formation [8, 9]. Increased cell adhesion mediated by these epithelial markers is suggested to be important for the activation of PI3K/AKT [10] and EGFR [11] pathways for anchorage-independent survival and proliferation, as well as for the invasion into local tissues via collective cell movement [12, 13]. We have previously identified overexpression of transcriptional corepressor protein, CtBP2, in ovarian cancer and its function in regulating cell growth and chemoresponse [14, 15], and also shown that CtBP2 is an oncogene that may play a significant role in epigenetic silencing of BRCA1 function in sporadic epithelial ovarian cancer [16]. C-terminal binding protein (CtBP) was originally identified as a protein that interacts with the C-terminal region of adenoviral oncoprotein E1A, which results in the reduced ability of E1A to transform cells [17, 18]. Mammalian CtBP family members include CtBP1 and CtBP2 isoforms, which carry diverse functions in embryogenesis and vertebrate development [19]. CtBP proteins promote cell survival by suppressing the expression of several pro-apoptotic genes, thus acting as apoptotic transcriptional regulators [20]. In addition, CtBPs promote cell survival through the maintenance of mitotic fidelity [21]. Loss of CtBP expression suppresses cell proliferation through a combination of apoptosis, reduction in cell cycle progression, and aberrations in transit through mitosis [21]. Alpatov *et al.* reported that CtBP1 interacts with a 140-kDa nucleoprotein named Pinin, which relieves CtBP1-mediated repression of E-cadherin expression [22]. Pinin was originally identified as an intermediate filament-associating protein in the desmosome complex [23] and was later found to co-exist in the nucleus [24]. Conditional disruption of Pinin expression in mice [25, 26] and in cell lines [27] resulted in cellular apoptosis and severe developmental problems.

In this study, we aimed to investigate the expression level of Pinin in ovarian tumors and its interactions with CtBP proteins in ovarian cancer cells. As Pinin has been implicated in alternative pre-mRNA splicing [28, 29], we also performed massively parallel paired-end RNA sequencing to explore the consequences of knocking down Pinin expression on gene transcription and RNA splicing variants.

## RESULTS

### Pinin is overexpressed in ovarian tumors and ovarian cancer cell lines

**Figure 1:**
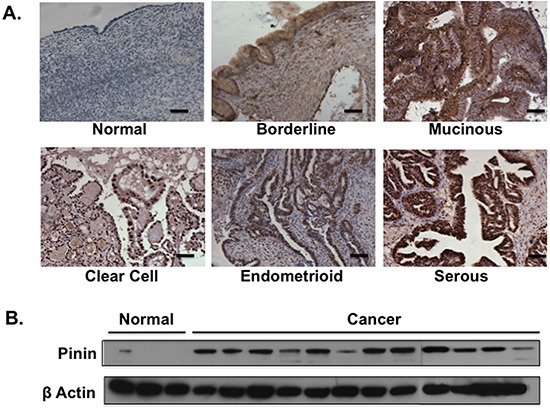
Pinin expression in clinical ovarian specimens and ovarian cell lines **A.** Representative of Pinin staining in clinical ovarian specimens. To highlight the tumor cell population, the slides were counterstained with hematoxylin (purple). The Pinin staining is in brown color. Scale bars represent 50μm. **B.** Western blot analysis of ovarian cell lysates for Pinin expression. β–actin was used as loading control.

We first investigated the expression pattern of Pinin in clinical ovarian specimens. A panel of normal ovary and, benign, borderline and invasive ovarian tumors (n=74) were subjected to immunohistochemistry (IHC) staining for Pinin (Figure 1A). ANOVA and post hoc analysis (Table 1) showed significant overexpression of Pinin (*p* < 0.001) in malignant and borderline tumors compared to normal ovaries. When the analysis was performed to evaluate the expression among different histologic subtypes within the invasive tumor group, the serous subtype showed relatively higher Pinin expression than the mucinous subtype (*p* = 0.003). We also performed Western blot analysis to evaluate the expression of Pinin in our panel of immortalized normal human ovarian surface epithelial (HOSE) cell lines and ovarian cancer cell lines. The results (Figure 1B) showed that Pinin was overexpressed in ten out of twelve ovarian cancer cell lines compared with normal HOSE cell lines. Hence, collectively, the results show that Pinin is overexpressed in most of the ovarian cancer cells.

**Table 1:** Diagnostic and histologic characteristics of Pinin expression in clinical ovarian specimens

Characteristics		Number of cases	Mean of scores	*P*-value
*Diagnostic*				
	Healthy	8	0.29	< 0.001
	Benign	6	3.00	
	Borderline	8	7.00	
	Invasive	52	6.89	
*Histology*				
	Serous	26	8.08	0.003
	Mucinous	14	5.18	
	Endometrioid	7	6.50	
	Clear Cell	5	6.00	

Notes for Tukey's post hoc analysis: Significant differences between normal and borderline and between normal and invasive tumors in Diagnostic; and significant difference between serous and mucinous tumors in Histologic.

### Pinin interacts with CtBP proteins in the nuclei of cancer cells

**Figure 2:**
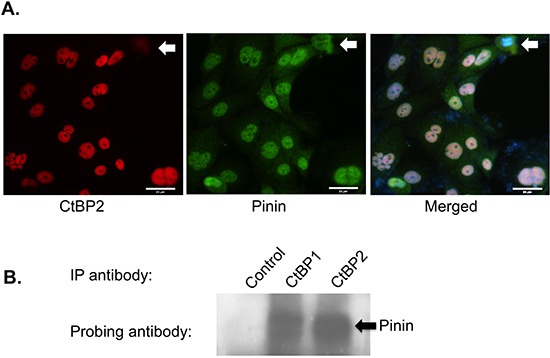
CtBP and Pinin interact with each other and are co-localized in the nuclei of cells **A.** Immunofluorescence to demonstrate the co-localization of CtBP2 (pseudo-colored in red) and Pinin (pseudo-colored in green) proteins in the nuclei of ovarian cancer cells. Shown on the right is the overlaid image of both red and green pictures together with blue DAPI DNA stain. The block arrow indicates the cells undergoing mitosis. Size bar represents 35μm. **B.** Co-immunoprecipitation assay using a mouse control antibody, CtBP1 antibody, and CtBP2 antibody, respectively. The immunoprecipitated lysates were fractionated, and the proteins were transferred to PVDF membrane and probed with an anti-Pinin antibody. The block arrow indicates the position of the Pinin protein band.

Pinin has been shown to interact with CtBP1 to act on E-cadherin promoter [30]. As we previously have shown that CtBP2 is overexpressed in ovarian cancer [14], it would be of interest to investigate whether CtBP2 also interacts with Pinin. Fluorescence microscopy of ovarian cancer cells stained with fluorescently labeled Pinin and CtBP2 antibodies showed that they were co-localized in the nuclei of the cells (Figure 2A), similar to the co-localization of CtBP1 with Pinin (data not shown). Interestingly, immunostaining also showed that whereas CtBP2 protein was lost in cells undergoing mitosis, Pinin protein remained in the cytosol of the cells (block arrow in Figure 2A). To further investigate the interaction between Pinin and CtBP proteins, co-immunoprecipitation was performed using CtBP1 and CtBP2 antibodies, respectively, to immunoprecipitate intracellular CtBP proteins. Western blot analysis of the immunoprecipitates showed that Pinin was co-immunoprecipitated with both CtBP proteins (Figure 2B). Hence, both immunofluorescence and co-immunoprecipitation assays suggest that Pinin physically associates with both CtBP1 and CtBP2 proteins in the nuclei of ovarian cancer cells.

### SKOV3-IP^Luc^ ovarian cancer cells with knockdown (KD) of Pinin expression showed deficiency in cell adhesion and other transformed phenotypes

**Figure 3:**
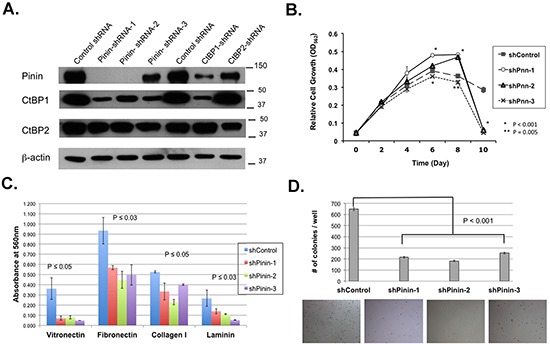
Ovarian cancer cells with Pinin knockdown showed reduction in CtBP1 expression and suppression of cell adhesion and clonogenecity **A.** Western blot analysis of Control, Pinin-KD, CtBP1-KD and CtBP2-KD cell lysates for the expression of Pinin, CtBP1, CtBP2. β–actin was used as loading control. **B.** Cell growth assay to determine the effect of Pinin knockdown on cell growth. **C.** Cell adhesion assay to compare the adhesion of control and Pinin-KD cell lines to different extracellular matrices. **D.** Soft agar assay to compare the clonogenecity of control and Pinin-KD cells. The bottom panel shows the representative images of the cell colonies after crystal violet staining. All the data shown are averages of triplicates of data from two independent experiments.

To explore the potential function of Pinin in ovarian cancer, we have established three knockdown SKOV3-IP^Luc^ ovarian cancer cell lines employing lentiviral particles harboring three different Pinin-targeting short hairpin RNA (shRNA) constructs. We compared the expression of Pinin, CtBP1, and CtBP2 in these three knockdown cell lines with the control SKOV3-IP^Luc^ cancer cell line, and a pair of SKOV3-IP^Luc^ ovarian cancer cell lines with knockdown of CtBP1 and CtBP2 expression, respectively. The result of the Western blot analysis (Figure 3A) shows that the three Pinin-KD cell lines together with both CtBP1-KD and CtBP2-KD cell lines had significant reduction of Pinin expression. However, a surprising observation is that the Pinin-KD cell lines also showed specific downregulation of CtBP1 expression, without significant changes of CtBP2 expression. Cell growth study did not reveal any significant growth hindrance of the Pinin knockdown cell lines. However, by day 10, all the three Pinin-KD cell lines showed a drastic reduction of MTT readings as compared with the control cell line (Figure 3B). We repeated the experiment together with CtBP1-KD and CtBP2-KD cell lines and also monitored the cells during the cell growth study (Supplementary Figure S1). By day 7, when the control cancer cells were becoming very confluent, all the Pinin and CtBP1 knockdown cell lines showed, with CtBP2-KD cells a lesser degree, excessive detachment from the culture plates. This phenomenon suggests a reduction of cell adhesion in the knockdown cancer cells, similar to what we have reported for the CtBP2-KD cancer cells [14]. The cell lines were then tested for the ability to attach on Cell Adhesion strips coated with different extracellular matrices. The results showed that the Pinin-KD cell lines adhered more poorly to different extracellular matrices than the control cells (Figure 3C).

We next tested the cell lines for anchorage-independent growth, a hallmark for transformed cells. The Pinin-KD cells formed only about 30-40% of the average number of colonies that the control line formed in the soft agar assay (Figure 3D). We also investigated whether the knockdown cells showed any differences in response to Paclitaxel, a drug used in the standard treatment regimen of ovarian cancer patients. The result (Supplementary Figure S2) showed that the Pinin-KD cell lines exhibited significantly higher sensitivity to the Paclitaxel treatment. In the meantime, CtBP1-KD and CtBP2-KD cell lines showed significant cell death only at very high doses of Paclitaxel, which we have reported previously [14].

Collectively, our functional studies illustrate the important function of Pinin in cell adhesion, clonogenicity, and chemoresponse.

### Next-generation RNA sequencing revealed that Pinin-KD and CtBP-KD cancer cells showed significant overlap of differential gene expression and RNA splicing aberrations

**Figure 4:**
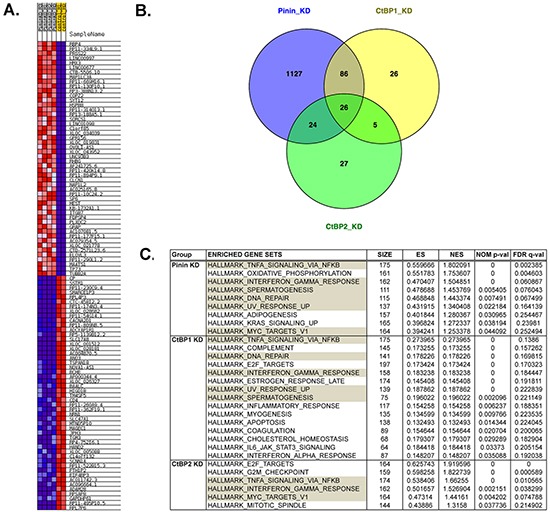
Analysis of RNA sequencing data at the gene level indicates the overlap between Pinin-KD cells and CtBP-KD cells in gene expression and cell function **A.** Top 50 up-regulated and top 50 down-regulated genes in Pinin-KD cells compared to control cells, **B.** Venn diagram to show the overlapping of differentially expressed genes among the three knockdown groups, and **C.** Significant gene set enrichments of differentially expressed genes for Pinin-KD, CtBP1-KD, and CtBP2-KD cancer cells determined by GSEA. The enriched gene sets of Pinin-KD cancer cells that are shared in the CtBP1-KD and CtBP2-KD cells are shaded in grey.

In order to understand the underlying mechanisms by which Pinin and CtBP regulate the phenotypes of ovarian cancer cells, gene expression profiling was performed. Because previous studies have implied that Pinin associates with RNA splicing factors and is involved in alternative pre-mRNA splicing [28, 29], we therefore have opted for next-generation RNA sequencing to determine gene expression and potential aberrations in alternative mRNA splicing. Ten total RNA samples, with two separate RNA preparations for each of the control, CtBP1-KD, CtBP2-KD, Pinin-KD1 and Pinin-KD2 cancer cell lines, were submitted for cDNA library preparation and massively parallel paired-end multiplex RNA sequencing and analysis as described in Materials and Methods. Analysis of the RNA sequencing data at the gene level revealed all the significant differentially expressed genes between control cells and the three knockdown groups, which are presented in Supplementary Table S1. Supplementary Table S1 also highlights a significant representation of noncoding RNAs including microRNAs, antisense RNAs, long intergenic noncoding RNAs (lncRNAs), and small nucleolar RNAs (snoRNAs) in Pinin-KD (21.5%), CtBP1-KD (24.5%), and CtBP2-KD (23.2%) cancer cell. The heatmap for the top 50 upregulated and top 50 downregulated genes between control and Pinin-KD cells is shown in Figure 4A. A Venn diagram (Figure 4B) was drawn to illustrate the overlapping differentially expressed genes among the three knockdown groups. There are 112 differentially expressed genes shared between Pinin-KD and CtBP1-KD cells, and less than half of that number (50) between Pinin-KD and CtBP2-KD cells. There are 26 genes that are shared by all three groups. The identities and the quantitative information of these 26 genes are listed in Table 2. In Table 2, quantitative information of two interesting genes that are significant only for Pinin-KD and CtBP1-KD cells but not in CtBP2-KD cells is also presented. One differentially expressed gene in both Pinin-KD and CtBP1-KD cells but not in CtBP2-KD cells is CtBP1. This mRNA finding corroborates the Western blot analysis result (Figure 3A). The second gene is epithelial splicing regulatory protein 1 (ESRP1), which has been reported to associate with Pinin in human corneal epithelial cells [29].

**Table 2:** Significant genes that are differentially expressed between control and the Pinin_KD and CtBP_KD groups

Gene Identifier	Locus	Gene	Log2(fold_change)		
			Pinin_KD	CtBP1_KD	CtBP2_KD
N/A	chr21:16070521-16627397	AP000962.1	8.48333	8.25676	6.94265
ENSG00000207863	chr21:16070521-16627397	MIR125B2	8.48333	8.25676	6.94265
ENSG00000207638	chr21:16070521-16627397	MIR99A	8.48333	8.25676	6.94265
ENSG00000215386	chr21:16070521-16627397	MIR99AHG	8.48333	8.25676	6.94265
ENSG00000199030	chr21:16070521-16627397	MIRLET7C	8.48333	8.25676	6.94265
ENSG00000162493	chr1:13583464-13617957	PDPN	6.37081	4.4568	3.95776
ENSG00000126561	chr17:42287546-42311943	STAT5A	5.3832	6.06392	6.11211
ENSG00000147872	chr9:19108374-19149290	PLIN2	5.00948	5.11097	4.40447
ENSG00000005243	chr17:48026166-48038030	COPZ2	4.66948	5.07214	3.75063
ENSG00000104413	chr8:94641073-94707466	ESRP1*	4.03037	4.46135	1.93086*
ENSG00000120915	chr8:27490778-27545564	EPHX2	3.72851	3.71745	2.76276
ENSG00000111859	chr6:11173451-11382348	NEDD9	3.6817	3.84812	4.00797
ENSG00000137673	chr11:102520507-102530753	MMP7	3.50457	5.20516	5.08387
ENSG00000173227	chr11:67006777-67050863	SYT12	3.50038	4.70088	5.12623
ENSG00000198715	chr1:156282934-156295689	C1orf85	2.91796	3.63491	2.78224
ENSG00000184292	chr1:58575422-58577773	TACSTD2	2.40479	3.39011	2.11716
ENSG00000182195	chrX:141175744-141177125	LDOC1	2.39325	3.44225	2.11582
ENSG00000132530	chr17:6755446-6776116	XAF1	2.05745	2.16076	2.3786
ENSG00000264230	chr10:46369086-46537864	ANXA8L1	1.77353	2.3488	2.32286
ENSG00000279458	chr10:46369086-46537864	CH17-335B8.4	1.77353	2.3488	2.32286
ENSG00000273225	chr10:46369086-46537864	FAM25BP	1.77353	2.3488	2.32286
N/A	chr10:46369086-46537864	HNRNPA1P33	1.77353	2.3488	2.32286
ENSG00000169129	chr10:114294823-114404756	AFAP1L2	1.66289	2.7914	2.49827
ENSG00000159692	chr4:1211447-1288291	CTBP1*	−2.09237	−2.83261	−0.139063*
ENSG00000160179	chr21:42199688-42297244	ABCG1	−1.94447	−2.71968	−3.46974
ENSG00000139116	chr12:39293227-39443390	KIF21A	−3.46638	−3.58121	−3.12557
ENSG00000252974	chr12:39293227-39443390	AC121334.1	−3.46638	−3.58121	−3.12557
ENSG00000108602	chr17:19737681-19748943	ALDH3A1	−5.02499	−4.20907	−2.53341

* Genes and differential expression that are not significant in the CtBP2_KD group.

Characterization of the differentially expressed genes of the Pinin-KD cells using Gene Set Enrichment Analysis (GSEA) demonstrated significant enrichment of gene sets in pathways for tumor necrosis factor alpha (TNFα) signaling mediated by transcription factor NFκB, interferon inflammation response, and DNA repair (Figure 4C and Supplementary Figure S3). The transcriptome of CtBP1-KD cells shares significant overlap of gene sets in the enriched pathways for Pinin-KD cells, suggesting that Pinin and CtBP1 are interacting for the similar cell function. The transcriptome of CtBP2-KD cells also had overlapping pathways with Pinin-KD cells. However, it appears that CtBP2 serves additional functions in G2M checkpoint and mitotic spindle, which are absent in both Pinin-KD and CtBP1-KD cells (Figure 4C).

**Figure 5:**
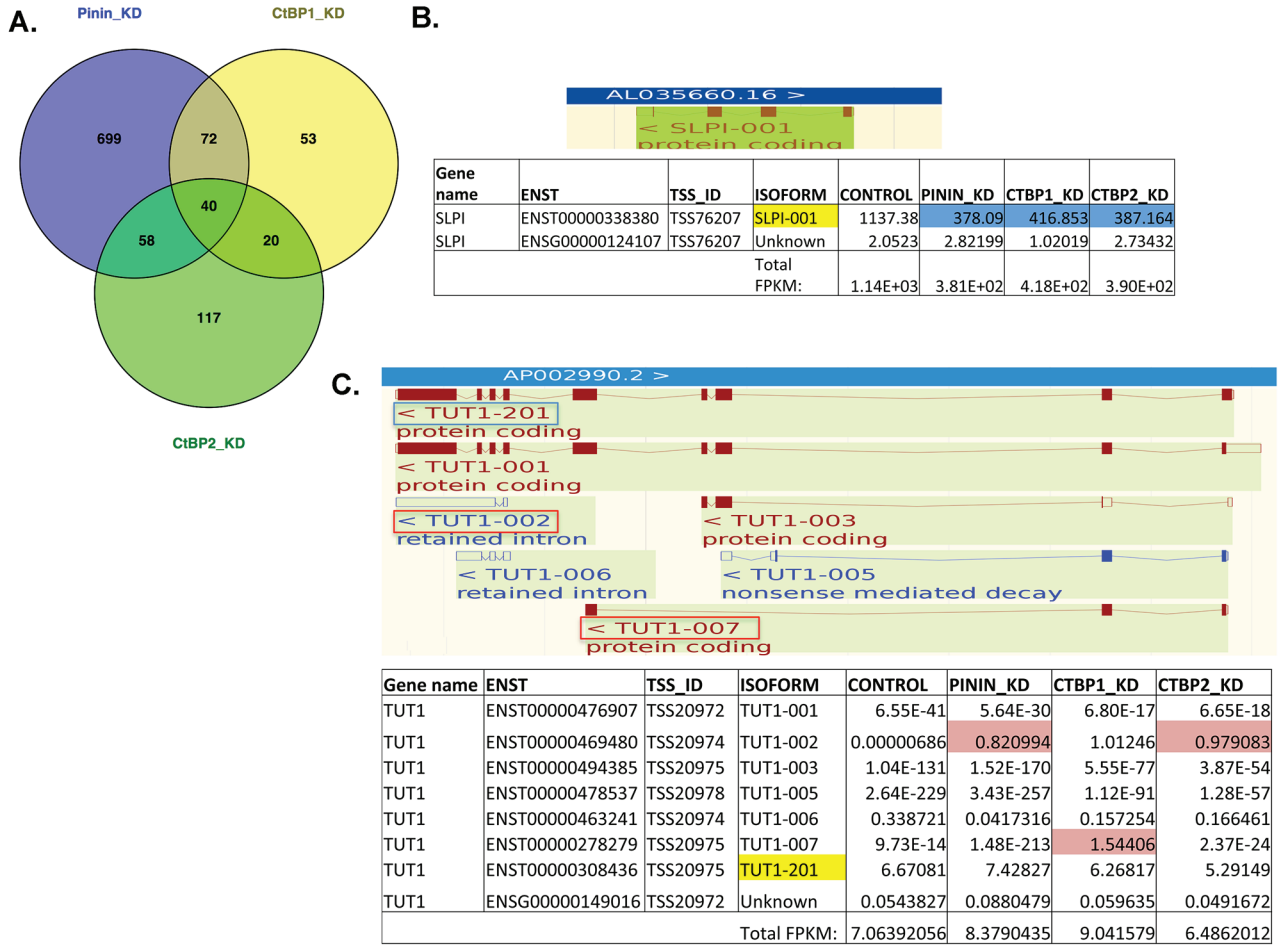
Analysis of RNA sequencing data at the transcript level identified significant transcript isoforms that could affect gene expression and gene function **A.** Venn diagram to show the overlap of differentially expressed isoforms among the three knockdown groups, **B.** the exon-intron structure of the known isoform (top) and the FPKM expression counts of the known and unknown isoforms of the gene SLPI in the different cell lines (bottom), and **C.** the exon-intron structure (top) and the FPKM expression counts of the different isoforms of the gene TUT1 in the different cell lines (bottom). For (B) and (C), the protein-coding isoforms are shown in brown, and the noncoding isoforms are shown in blue in the top panels. The discussed TUT1 isoforms are also bracketed in (C). In the bottom panels, the protein-coding isoforms are shaded in yellow, and the significantly upregulated isoforms are shaded in pink, downregulated isoforms are shaded in blue.

In order to investigate the potential effects of gene knockdown on RNA alternative splicing or alternative transcription start site (TSS) selection, the RNA data was analyzed at transcript level. Supplementary Table S2 lists all the significant differentially expressed transcripts between control cells and the three knockdown groups. The Venn diagram (Figure 5A) shows that the number of genes with significant differentially expressed transcripts shared between Pinin-KD and CtBP1-KD cells (112) is similar to the number of genes shared between Pinin-KD and CtBP2-KD cells (98). There are 40 genes that are shared by all three knockdown groups for differentially expressed transcripts (Table 3). Most of the transcripts listed in Table 3 were significant in all of the three knockdown groups, and for the genes such as MMP7, INHBA, and HSPA1A with upregulated transcripts, these genes were also significantly upregulated at the gene level (Table 2). For the genes that had significantly downregulated transcripts, they either had a small number of alternatively spliced transcripts similar to the genes with upregulated transcripts, or the genes had moderate to larger number of transcript isoforms. One example for the genes with a small number of alternatively spliced transcripts is Secretory Leukocyte Peptidase Inhibitor (SLPI) (Figure 5B), whose gene product is a well-studied serum biomarker for ovarian cancer [31, 32]. The examples for the moderate to large number of alternative isoforms are FGFRL1 (Supplementary Figure S4) and CtBP1 (Supplementary Figure S5). The significant differentially expressed isoforms of all these genes were the same predominantly expressing protein-coding isoforms and therefore contributed to the changes of the total expression units of the genes in the knockdown groups, as defined by Fragments Per Kilobase of exon per Million fragments mapped (FPKM).

**Table 3:** Significant transcript isoforms that are differentially expressed between control and the Pinin_KD and CtBP_KD groups

Transcript Identifier	TSS_ID	Gene	Locus	Log2(fold_change)		
				Pinin_KD	CtBP1_KD	CtBP2_KD
ENST00000469480	TSS20974	TUT1*	chr11:62559600-62592177	16.8671	17.1921*	17.1437
ENST00000242208	TSS109160	INHBA	chr7:41667167-41779388	3.65232	3.0042	3.92722
ENST00000278742	TSS19285	ST14	chr11:130159561-130210376	3.52693	4.14332	2.15435
ENST00000260227	TSS22249	MMP7	chr11:102520507-102530753	3.49994	5.19935	5.08377
ENST00000608703	TSS100382	HSPA1A	chr6:31815463-31817946	2.76283	2.94962	1.57709
ENST00000370526	TSS124554	LDOC1	chrX:141175744-141177125	2.44441	3.33245	2.44022
ENST00000371225	TSS7656	TACSTD2	chr1:58575422-58577773	2.40479	3.39011	2.11716
ENST00000375650	TSS100383	HSPA1B	chr6:31827734-31830255	2.34891	2.34832	1.32126
ENST00000287590	TSS69146	B3GNT7	chr2:231395542-231401164	2.33953	2.01437	1.81141
ENST00000395748	TSS89593	AREG	chr4:74445133-74455009	2.31289	2.67371	1.52912
ENST00000392452	TSS88162	MB21D2	chr3:192796814-192918161	2.24372	1.72794	1.99704
ENSG00000249306	TSS96391	LINC01411	chr5:174336294-174532457	2.17866	1.8394	1.875
ENST00000294435	TSS376	RBP7	chr1:9997205-10016020	2.12295	2.34135	2.34447
ENST00000304129	TSS15105	AFAP1L2	chr10:114294823-114404756	1.81005	2.61546	2.61908
ENST00000497571	TSS13349	KLF6	chr10:3775995-3785281	1.69485	1.42906	1.1668
ENST00000257836	TSS16395	PRRG4	chr11:32829942-32858123	1.59563	1.50225	1.65308
ENST00000309166	TSS19897	NRIP3	chr11:8980575-9004049	1.54851	1.78921	1.35272
ENST00000196371	TSS97281	OXCT1	chr5:41730064-41872241	1.4849	1.77312	1.73092
ENST00000263464	TSS18533	BIRC3	chr11:102317449-102339403	1.3607	1.63618	1.60977
ENST00000216117	TSS79110	HMOX1	chr22:35380360-35394214	1.29473	1.85747	−1.13128
ENST00000368223	TSS9315	NES	chr1:156668762-156677397	1.23924	1.69318	2.23559
ENST00000451311	TSS120936	TMSB4X*	chrX:12975107-12977227	1.11634	0.738397*	0.588678*
ENST00000348367	TSS15083	GPAM	chr10:112149863-112215377	1.03841	1.37058	1.69528
ENST00000339276	TSS1000	SFN	chr1:26863137-26864457	0.918878	0.989102	1.21727
ENST00000449283	TSS122506	SPANXB2	chrX:141002590-141003706	−0.98586	−1.07802	−1.42392
ENST00000369783	TSS15002	CALHM3	chr10:103472803-103479240	−1.17387	−1.17278	−1.97225
ENST00000369409	TSS3009	PHGDH	chr1:119659797-119744215	−1.46291	−1.93556	−3.10031
ENST00000258829	TSS35372	NKX2-8	chr14:36580578-36582607	−1.58188	−1.97299	−1.65747
ENST00000338380	TSS76207	SLPI	chr20:45252238-45254564	−1.58891	−1.42436	−1.53095
ENST00000264748	TSS88471	FGFRL1	chr4:1009935-1026897	−1.69614	−1.08733	−1.80221
ENST00000358321	TSS78736	SUSD2	chr22:24181258-24189110	−1.71507	−3.04531	−2.35938
ENSG00000234593	TSS6195	RP4-704D23.1	chr1:14348954-14351023	−1.92077	−3.41104	−2.25659
ENST00000354666	TSS102549	ELOVL2	chr6:10980758-11078226	−2.14391	−3.03229	−1.70635
ENST00000319211	TSS94611	F2R	chr5:76403248-76735781	−2.17424	−2.63607	−1.23504
ENST00000555004	TSS34229	C14orf132	chr14:96039323-96094080	−2.41648	−2.55822	−1.39087
ENST00000217939	TSS122913	MXRA5	chrX:3308564-3346641	−4.14304	−2.25727	−1.66432
ENSG00000234626	TSS80398	RP1-149A16.12	chr22:32327170-32343105	−4.31484	−2.81994	−2.53571
ENST00000439647	TSS84273	AP2M1*	chr3:184134018-184684758	−15.7981	0.015061*	−0.84884*
ENST00000503081	TSS99619	GNB2L1*	chr5:181236908-181272307	−66.6096	−0.601417*	−25.4927
ENST00000523108	TSS99522	MGAT4B*	chr5:179797596-179907859	−167.305	0.813388*	−89.4254

* Genes and expression changes that are not from the same ENST isoforms.

In Table 3, there were also genes such as AP2M1, GNB2L1, and MGAT4B that had significantly downregulated isoforms not shared by all the knockdown groups. As illustrated by MGAT4B in Supplementary Figure S6, these genes had a very large number of isoforms, and the significantly altered isoforms were not the predominantly expressing protein-coding isoforms and did not affect the total FPKM expression of the genes. On the other hand, for the gene encoding nucleotidyl transferase terminal uridylyl transferase 1 (TUT1) that had the isoform (TUT1-002) with the highest differential fold-change in the knockdown groups (Table 3), this isoform and the other isoform that was significantly upregulated in CtBP1-KD cells, TUT1-007, were also not the major protein-coding transcript, which was TUT1-201 (Figure 5C). However, isoform TUT1-002 is a noncoding transcript with intron retention, and isoform TUT1-007 encodes a peptide sequence only for the first 165 amino acids of the wild-type gene product, which lacks a PAP/25A-associated domain important for polynucleotide adenylyltransferase activity. Hence, both of these two upregulated isoforms appear to be nonfunctional and would significantly contribute to the increase of the nonfunctional isoform pool (Figure 5C), which might negatively affect the function of the gene.

## DISCUSSION

One characteristic of ovarian tumors is that they express many epithelial proteins [2–5]. Ectopic expression of E-cadherin, an epithelial marker, in normal ovarian surface epithelial cells caused mesenchymal-epithelial transition (MET) *in vitro* and tumor formation in a mouse model [8, 9]. The epithelial phenotype of ovarian tumors facilitates the activation of PI3K/AKT [10] and EGFR [11] pathways for tumor growth and survival and also for the invasion into local tissues via collective cell movement [12, 13]. Pinin has shown its importance in maintaining epithelial cell identity. Pinin depletion caused apoptosis and reduced survival of cells *in vitro* [27] and conditional knockout of Pinin caused defects in mouse corneal epithelial cell differentiation [25] and intestine morphogenesis [33]. In our study, we showed strong expression of Pinin in many ovarian tumors and ovarian cancer cell lines. Knockdown of Pinin expression in ovarian cancer cells resulted in significant reduction in cell adhesion, anchorage-independent growth, and increased sensitivity to the chemotherapeutic agent Paclitaxel. The results of the functional studies collectively indicated that Pinin, resembling other epithelial markers such as E-cadherin [8, 9], is important in ovarian tumorigenesis and progression.

Our characterization also indicates that Pinin interacts with both human CtBP1 and CtBP2 proteins in the nuclei of ovarian cancer cells. Interestingly, we observed that CtBP2 was absent in the mitotic cells, while Pinin was still present in the cytosol (Figure 2A). It is likely that CtBP2 expression is cell cycle-regulated and is related to its additional function in G2/M checkpoint and spindle regulation, as suggested by the GSEA analysis of the RNA sequencing data (Figure 4C). Another finding about the interaction between Pinin and CtBP proteins is that Pinin levels were reduced in both CtBP1-KD and CtBP2-KD cancer cells. In contrary, only CtBP1 expression, not CtBP2 expression, was declined in the Pinin-KD cells. This was confirmed in the RNA-sequencing analysis, which clearly demonstrated that the major protein-coding isoform and the total RNA expression of the gene were suppressed in the Pinin-KD cells (Supplementary Figure S5). The mutual suppression of the first gene's expression in the second gene's knockdown cell lines between Pinin and CtBP1 suggests the existence of a feedback loop in regulating one another's expression. Besides co-localization and mutual regulation of each other's expression, the intimate relationship between Pinin and CtBP1 is also reflected in the larger number of co-regulated genes (Figure 4B) and overlapping enriched gene sets (Figure 4C) between Pinin-KD and CtBP1-KD cell lines than between Pinin-KD and CtBP2-KD cell lines. The most significant pathways enriched in Pinin-KD and CtBP1-KD cells include the canonical NFκB signaling pathway induced by TNFα and interferon response pathway. It is well known that these two conserved cytokine pathways are involved in the response of innate immunity to inflammation caused by physiological and oxidative stress, while chronic inflammation is pro-tumorigenic and NFκB is constitutively activated in many types of cancer to upregulate anti-apoptotic genes [34–36]. In combination with the DNA repair pathway, the enrichment of the differentially expressed genes in inflammation pathways might indicate the stress response of the cancer cells to gene knockdown. These survival response pathways were also enriched in the CtBP2-KD cells. However, as stated before, CtBP2 knockdown also provoked a response to cell cycle perturbation (Figure 4C).

Deregulated RNA metabolic mechanisms, especially in RNA splicing, are underappreciated in the field of cancer research. However, there is increasing evidence to support the notion that deregulations in RNA metabolism are associated with cancer development and its phenotypes [37–39]. By examining the global adenosine-to-inosine RNA editing profiles of 6,236 patients samples from the Cancer Genome Atlas, Han *et al.* identified myriad clinically relevant altered RNA-editing events, many of which are in the noncoding regions, that can affect cell viability and drug sensitivity [39]. In a study to look up subtype-specific differentially spliced genes and splicing isoforms, Eswaran *et al.* have revealed RNA splicing signatures for triple-negative breast cancer (TNBC), non-TNBC, and HER2-positive breast cancer [38]. In another breast cancer study to employ a Bayesian model to characterize hundreds of deregulated alternative splicing events mediated by the splicing factor SRSF1 that is overexpressed in this cancer type, Ancuzuków *et al.* reported the positional effects of SRSF1 binding on cassette exons on the splicing results [37]. To this end, Pinin has been implicated in alternative pre-mRNA splicing [28, 29], and a Pinin loss of function study has shown the alternative splicing patterns of SRSF1 [27].

In our analysis of the RNA sequencing data for Pinin-KD cancer cells, significant aberrations of RNA processing were found. Although the RNA sequencing methodology we used was not intended to look at noncoding RNAs in ovarian cancer cells, we found significant representation of poly(A)-tailed noncoding RNAs such as lincRNAs and pre-miRNAs in the significantly altered gene list (Table 2). There are also significant changes of splicing variants in the knockdown groups (Figure 5 and Supplementary Figures S4 and S6). Given the intimate interactions between Pinin and CtBP proteins, it is not surprising that the RNA processing aberrations were also observed in CtBP1-KD and CtBP2-KD cancer cells. In order to explore the function of Pinin and CtBP proteins on RNA alternative splicing, we primarily examined the significantly altered gene expression and RNA isoforms in all three knockdown cell lines. For most of the differentially expressed genes, the altered isoforms are the predominantly expressing protein-coding transcripts and their changes affect the ultimate expression of the target genes, which might reflect the direct effects of Pinin and CtBP proteins on gene transcription. However, there are also genes like AP2M1, GNB2L1, and MGAT4B that are associated with significantly downregulated isoforms, and some genes such as CD44, CTNND1, and ENAH whose alternative splicing forms have previously been described to associate with Pinin defects [29], the common feature of these genes is that they produce a large number of splicing variants, and the altered isoforms are not the predominantly expressing protein-coding transcripts. More validation studies are required to determine whether these altered splicing variants are only spurious RNAs or they have specific meaning on gene function.

Another outcome of this comprehensive analysis of the whole alternative splicing transcriptomes in Pinin-KD and CtBP-KD cells is that we identified significant changes in some genes that have been associated with RNA processing mechanisms. One gene was ESRP1, which has been found to be associated with Pinin in a previous study [29] and whose expression was significantly upregulated in Pinin-KD and CtBP1-KD cancer cells in the present study (Table 2). A new RNA-processing gene identified in this study was TUT1 (Table 3 and Figure 5C). TUT1 is a nucleotidyl transferase that functions as a terminal uridylyltransferase for small nuclear RNAs (snRNAs) such as U6 snRNA [40], and as a poly(A) polymerase that creates and cleaves the 3′-poly(A) tail of specific mRNA [41, 42], and recent studies have shown that TUT1 is a global regulator of miRNA abundance [43, 44] and regulates cell proliferation and other cell functions. In our in-silico analysis, we identified interesting overexpressing isoforms for this gene in the knockdown cell lines, which either encodes a noncoding transcript with intron retention (TUT1-002), or an N-terminal coding transcript (TUT1-007) that contains the RNA-binding RNA Recognition Motif (RRM) domain but lacks the PAP/25A-associated domain important for polynucleotide adenylyltransferase activity (Figure 5C). As these two RNA isoforms are the highest significantly expressing isoforms in the knockdown cancer cell lines (Table 3), it would be of great interest to determine whether an overexpression of these nonfunctional transcripts in the transcript pool would significantly affect gene function and cell phenotype.

In summary, we have shown significant overexpression of Pinin in ovarian tumors and its function in cell adhesion, clonogenicity, and drug response. Pinin is an important regulator in epithelial cell identity. As a recent article shows the function of CtBP2 in epigenetic reprogramming of cells for lineage commitment [45] and the serous subtype expressed a higher level of Pinin than the mucinous subtype of ovarian tumors in our study (Table 1), Pinin and CtBP proteins may interact with each other to regulate proliferation and local invasion of epithelial ovarian cancer cells and histologic lineage differentiation. In addition, our whole transcriptomic analysis of Pinin and CtBP knockdown cancer cells provides the first comprehensive portrait of significant transcriptional and splicing variants. It would be of paramount importance to study the myriad interactions among the RNA processing or editing proteins such as Pinin, CtBP, ESRP1, SRSF1, and TUT1, and identify and validate the key altered RNA isoforms resulted from the interactions and their impacts on the functions of ovarian cancer cells.

## MATERIALS AND METHODS

### Clinical specimens and ovarian cell lines

Archived specimens were obtained from patients with an IRB approved protocol at Brigham and Women's Hospital in Boston. All the surgical specimens were collected with patient consents. The immortalized normal human ovarian surface epithelial (HOSE) cell lines and ovarian cancer cell lines have been described before [46]. They were maintained in medium 199 and MCDB 105 (Sigma-Aldrich, Natick, MA, USA) (1:1) supplemented with 10% FCS.

### Immunohistochemistry

Immunohistochemistry (IHC) was performed on a panel of 74 archived formalin-fixed, paraffin-embedded tissues, including 8 normal ovarian tissues and 66 benign, borderline, and malignant ovarian tumors. Standard xylene deparaffinization, rehydration with a descending series of ethanol solutions, antigen retrieval (Vector Laboratories, Burlingame, CA), and blocking of endogenous peroxidases in 0.3% H_2_O_2_ were performed. The mouse anti-Pinin monoclonal antibody has been described before [22]. 3, 3 –diaminobenzidine (DAB) horseradish peroxidase substrate kit was used for color development (Vector Laboratories, Burlingame, CA). Staining was graded semiquantitatively by multiplying the proportion of the stained epithelial area (from 0 for absence to 3 for more than 95% of the total epithelial area) with the intensity of the stain (from 0 for negative staining to 3 for strongly positive staining).

### Establishment of ovarian cancer cell lines with respective knockdown (KD) of expression of Pinin, CtBP1, or CtBP2, and subsequent functional assays

Mission™ lentiviral gene-targeting and non-target control lentiviral shRNA constructs were purchased from Sigma-Aldrich (Natick, MA, USA) and the production of transduction particles and infection of SKOV3-IP^Luc^ ovarian cancer cells were performed according to manufacturer's protocol. The CtBP2 knockdown cancer cell line has been described previously [14]. The TRC numbers for the knockdown of CtBP1 and Pinin are: TRCN0000013738 for CtBP1 and TRCN0000072278, TRCN0000072279, and TRCN0000072280 for Pinin. Knockdown of gene expression in the resultant cell lines was confirmed by Western blot analysis.

Cell growth study was performed using methylthiazol tetrazolium (MTT) solution (5 mg/mL in PBS, Sigma-Aldrich, Natick, MA). Absorbance at 562 nm was determined on an ELx800 absorbance microplate reader (Bio-Tek, Winooski, VT). Cell adhesion assay was performed using the cell adhesion strips from Millicoat™ Screen kit ECM205 (EMD Millipore, Billerica, MA). For the experiment, 1×10^4^ cells were seeded to the strip wells and allowed to incubate for 1 hour. Nonadherent cells were washed away by phosphate buffered saline and the attached cells were stained using 0.2% crystal violet. The stain was solubilized in a 50:50 mixture of 0.1M NaH_2_PO, pH 4.5 and 50% ethanol and read at 562 nm.

For soft agar colony formation assay, 10^4^ single cells in medium were mixed with equal volume of pre-warmed 0.66% SeaPlaque™ agarose (Lonza, Allendale, NJ) and poured over a 0.5% agarose layer in wells of a six-well plate. After about 21 days, cell colonies were fixed with 10% methanol and 1% acetic acid and stained with 0.005% crystal violet (Sigma-Aldrich, Natick, MA) and counted using a dissecting microscope. Cellular sensitivity to Paclitaxel (Sigma-Aldrich, Natick, MA) was measured by treating the cells to the drugs for 48 hours and cell survival was estimated using MTT assays. All the functional assays were performed in triplicates and repeated twice.

### Immunofluorescence microscopy and co-immunoprecipitation assay

Wild-type SKOV3-IP^Luc^ ovarian cancer cells were fixed in 4% paraformaldehyde (Sigma-Aldrich, Natick, MA) and permeabilized using 0.5% Triton X-100. After blocking with 10% fetal bovine serum (Thermo Fisher Scientific, Waltham, MA), mouse monoclonal antibodies targeting CtBP1 or CtBP2 proteins (BD Biosciences, San Jose, CA) and a rabbit antibody targeting Pinin (Bethyl Laboratories, Montgomery, TX) were added and incubated for 2 hours. Alexa Fluor 647-conjugated anti-mouse and Alexa Fluor 546-conjugated anti-rabbit secondary antibodies (Thermo Fisher Scientific, Waltham, MA) were used to stain Pinin and the CtBP proteins, respectively. The stained cells were counterstained with DAPI (Thermo Fisher Scientific, Waltham, MA). Microscopic images were captured by a Leica DM IRE2 fluorescence microscope (Leica Microsystems, Bannockburn, IL) and analyzed by the OpenLab Cell Imaging System software (Leica Microsystems, Bannockburn, IL).

For preparing cell extracts enriched with nuclear proteins for Co-immunoprecipitation assays, cells were lysed by undergoing three freeze/thaw cycles in 200μl of Buffer A (200mM Tris, pH8.0, 0.3M KCl, 5mM MgCl_2_, 0.1% Tween 20, 10% glycerol, 10mM mercaptoethanol, and 0.2mM of phenylmethylsulfonyl fluoride) and on ice for 30 min. 400μl of Buffer B (same ingredients as Buffer A except for the absence of KCl) were added and the mixture was centrifuged at 16,000 rpm for 30 min. Equal amounts of the cell extracts were incubated overnight with the CtBP1 and CtBP2 antibodies (BD Biosciences, San Jose, CA), and control mouse IgG, respectively. The immune complex was captured by protein A/G immobilized on agarose beads (Thermo Fisher Scientific, Waltham, MA). After extensive washes, the immune complex proteins were fractionated by standard SDS-PAGE, transferred to PVDF membrane (Thermo Fisher Scientific, Waltham, MA) and analyzed by Western blot.

### Next-generation RNA sequencing and data analysis

Total RNA was extracted from the ten ovarian cancer cell lines using TRIzol reagent (Thermo Fisher Scientific, Waltham, MA). For cDNA library preparation, 500ng of total RNA with 260/280 OD greater than 1.8 were processed with KAPA Stranded mRNA-Seq Kit from Kapa Biosystems (Wilmington, MA) for mRNA purification and fragmentation, A-tailing, adapter ligation, and library amplification. During the adapter ligation step, 0.6μM NEXTflex™ RNA-Seq Barcode adaptors with different indexes (Bioo Scientific, Austin, TX) were ligated to the samples to provide unique barcodes for each of the ten libraries for multiplex sequencing. The amplified libraries were cleaned up using Agencourt AMPure XP beads (Beckman Coulter, Indianapolis, IN) and eluted in 20μL of 10 mM Tris buffer, pH 8. The quality of the libraries was tested on an Agilent 2100 Bioanalyzer (Agilent Technologies, Santa Clara, CA) and the concentrations of the libraries were determined by qPCR using KAPA Library Quantification Kit (Kapa Biosystems, Wilmington, MA). Ten libraries were pooled together to give a final library concentration of 17.7nM and a 50-bp paired-end sequencing run was performed on an Illumina HiSeq-2500 instrument (Illumina, San Diego, CA) in High-Output mode.

For the bioinformatics, HiSeq FASTQ data together with QC files were analyzed to ensure that no run-related problems occurred. Clean, adaptor-trimmed reads were aligned onto the human genome GRCh38 (accession GCA_000001405.15) using STAR v2.4.1b [47], a spliced aligner for RNA-seq reads. Transcriptome assembly and differential expression testing was performed using Cufflinks v2.2.1 [48]. Significant genes and transcripts were identified based on *p* < 0.05, as described by Eswaran *et al*. [49]. GENCODE v21 was used as the known transcript annotation database. The mask file for the tRNA, rRNA, and mitochondrial genes were generated using UCSC Table Browser. Fragments per kilobase of exon per million fragments mapped (FPKM) was employed as the RNA-sequencing expression unit for different transcript isoforms and for gene expression comparison. GSEA and Molecular Signature Database (MSigDB) v5.0 [50] were used for gene set enrichment analysis and heatmap generation. The GenePattern [51] module Read_group_trackingToGct v0.15 was used to convert the sequencing fragment counts of the genes from the transcriptome assembly into a file format suitable for input to GSEA. Venn diagrams were drawn using VENNY v2.0 (http://bioinfogp.cnb.csic.es/tools/venny/).

### Statistical analysis

All calculations were performed with MINITAB statistical software (Minitab, State College, PA) unless otherwise indicated. ANOVA with post hoc Tukey's multiple comparisons test was used to determine any significant differences of immunohistochemistry scores between groups. For the functional assays, significance of differences was determined using 2-tailed T-Test, with *P*-value less than 0.05 was considered statistically significant.

## SUPPLEMENTARY FIGURES AND TABLES






