# Correction: Pinin interacts with C-terminal binding proteins for RNA alternative splicing and epithelial cell identity of human ovarian cancer cells

**DOI:** 10.18632/oncotarget.15321

**Published:** 2017-02-13

**Authors:** Yanli Zhang, Jamie Sui-Lam Kwok, Pui-Wah Choi, Minghua Liu, Junzheng Yang, Margit Singh, Shu-Kay Ng, William R. Welch, Michael G. Muto, Stephen KW Tsui, Stephen P. Sugrue, Ross S. Berkowitz, Shu-Wing Ng

**Present**: Due to an omission on the part of the author, the affiliations of the first author are incomplete.

**Corrected**: Additional affiliation information for the first author is listed below. The authors sincerely apologize for this error.

Original article: Oncotarget. 2016; 7(10):11397-11411. doi: 10.18632/oncotarget.7242.

**PRESENT LIST:**

**Yanli Zhang^1^, Jamie Sui-Lam Kwok^2^, Pui-Wah Choi^1^, Minghua Liu^2^, Junzheng Yang^1^, Margit Singh^1^, Shu-Kay Ng^4^, William R. Welch^5^, Michael G. Muto^1^, Stephen KW Tsui^2^, Stephen P. Sugrue^3^, Ross S. Berkowitz^1^, Shu-Wing Ng^1^**

**^1^** Laboratory of Gynecologic Oncology, Division of Gynecologic Oncology, Department of Obstetrics, Gynecology and Reproductive Biology, Harvard Medical School, Boston, MA, USA

**^2^** School of Biomedical Sciences, The Chinese University of Hong Kong, Hong Kong

**^3^** Department of Anatomy and Cell Biology, University of Florida College of Medicine, Gainesville, FL, USA

**^4^** School of Medicine and Menzies Health Institute Queensland, Griffith University, Meadowbrook, Australia

**^5^** Department of Pathology, Brigham and Women’s Hospital, Harvard Medical School, Boston, MA, USA

**UPDATED LIST:**

**Yanli Zhang^1,6^, Jamie Sui-Lam Kwok^2^, Pui-Wah Choi^1^, Minghua Liu^2^, Junzheng Yang^1^, Margit Singh^1^, Shu-Kay Ng^4^, William R. Welch^5^, Michael G. Muto^1^, Stephen KW Tsui^2^, Stephen P. Sugrue^3^, Ross S. Berkowitz^1^, Shu-Wing Ng^1^**

**^1^** Laboratory of Gynecologic Oncology, Division of Gynecologic Oncology, Department of Obstetrics, Gynecology and Reproductive Biology, Harvard Medical School, Boston, MA, USA

**^2^** School of Biomedical Sciences, The Chinese University of Hong Kong, Hong Kong

**^3^** Department of Anatomy and Cell Biology, University of Florida College of Medicine, Gainesville, FL, USA

**^4^** School of Medicine and Menzies Health Institute Queensland, Griffith University, Meadowbrook, Australia

**^5^** Department of Pathology, Brigham and Women’s Hospital, Harvard Medical School, Boston, MA, USA

**^6^** Department of Obstetrics and Gynecology, Tongji Hospital, Tongji Medical College, Huazhong University of Science and Technology, Wuhan, China

