# Supplementary material for: Pinin interacts with C-terminal binding proteins for RNA alternative splicing and epithelial cell identity of human ovarian cancer cells
Source: Oncotarget. 2016 Feb 8;7(10):11397–411. doi: 10.18632/oncotarget.7242 (PMC4905481; doi:10.18632/oncotarget.7242)
Supplement: Supplementary file 1 [file oncotarget-07-11397-s001.pdf]

SUPPLEMENTARY FIGURES AND TABLES

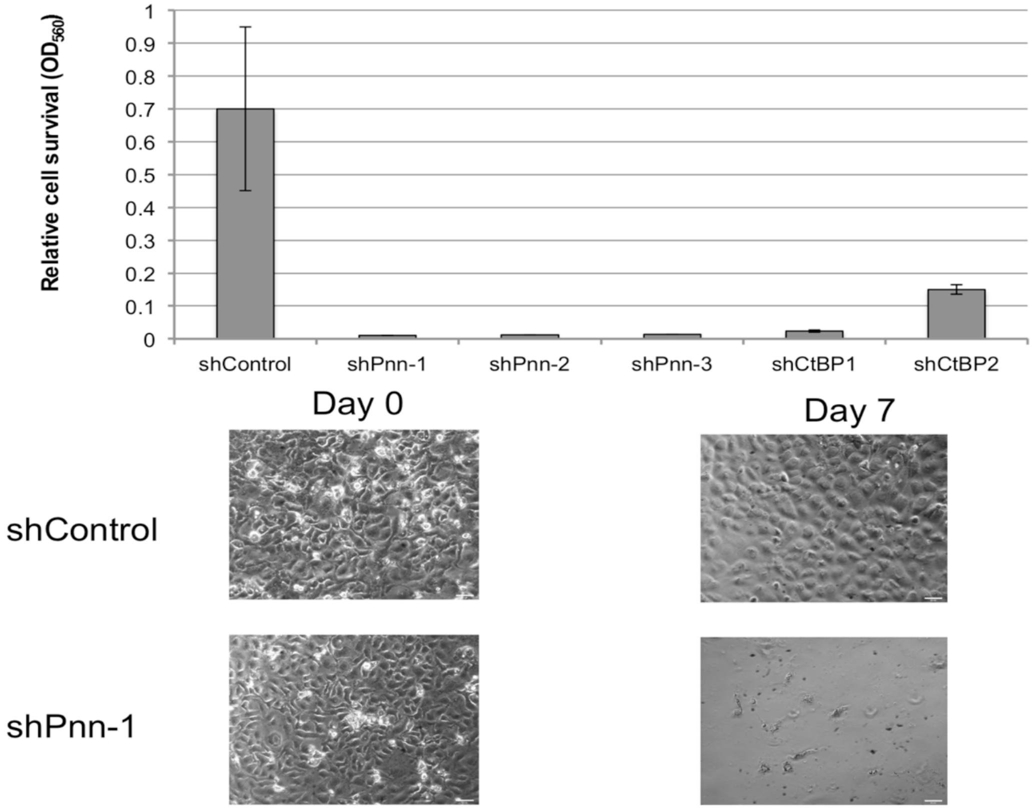

Supplementary Figure S1: Ovarian cancer cells with Pnn-Knockdown showed reduced survival at confluence. Control SKOV3-IP^{Luc} ovarian cancer cells and cancer cells with knockdown of Pinin, CtBP1, or CtBP2 expression were seeded and allowed to grow until Day 7. Images were taken on Day 0 and Day 7, when the cells were washed twice with PBS and MTT assay was performed to show the viability of the remaining cells.

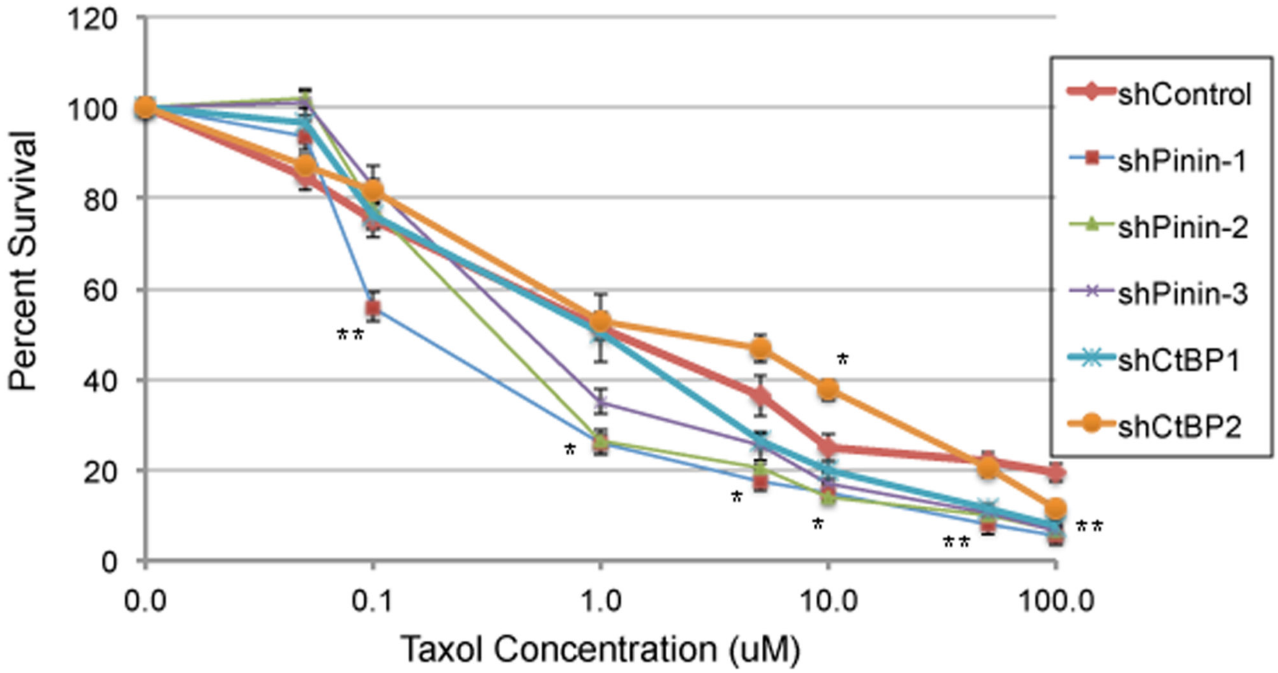

Supplementary Figure S2: Ovarian cancer cells with Pnn-Knockdown showed increased sensitivity to Paclitaxel. Control SKOV3-IP^{Luc} ovarian cancer cells and cancer cells with knockdown of Pinin, CtBP1, or CtBP2 expression were treated with different concentrations of Paclitaxel for 48 hours and MTT assay was performed to determine the survival. * $p < 0.05$, ** $p < 0.01$.

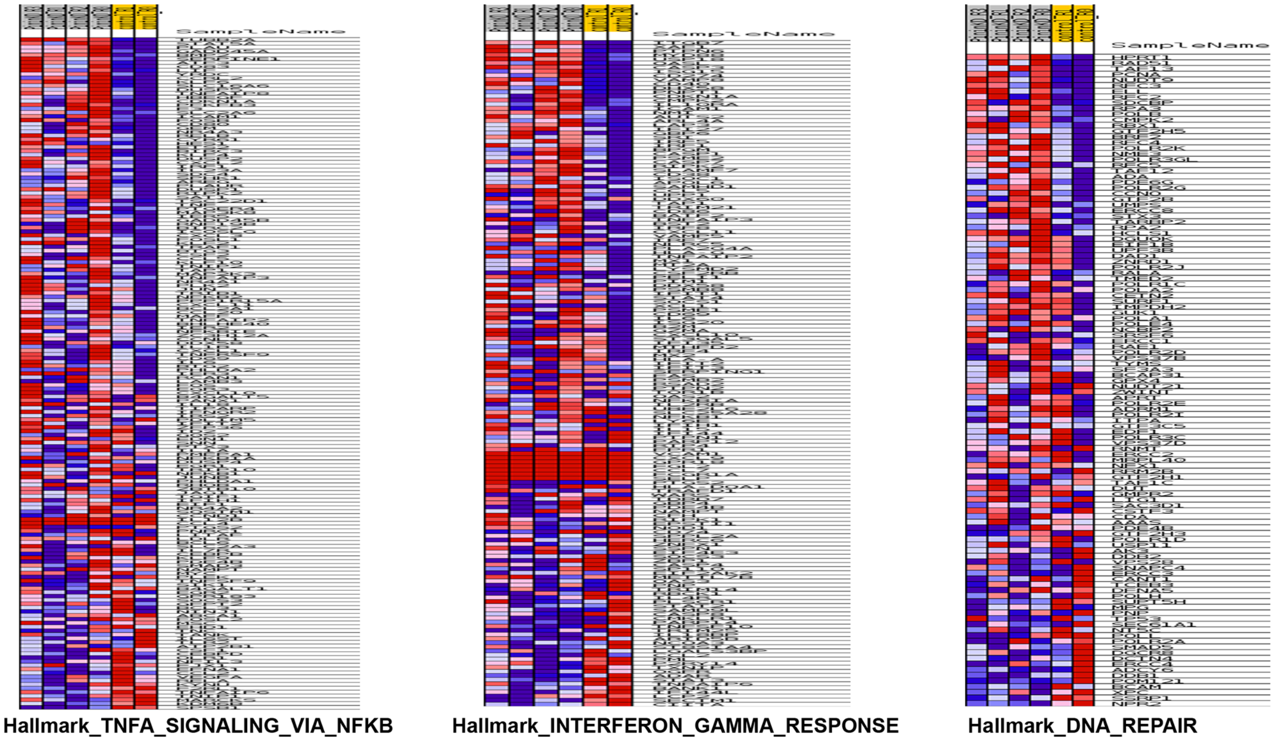

Supplementary Figure S3: Heat maps showing differentially expressed genes for enriched pathways identified by GSEA in MSigDB. Shown here are the heatmaps for 3 top pathways enriched for differentially expressed genes between Control and Pinin_KD ovarian cancer cell lines identified by GSEA in the Hallmark pathway collection.

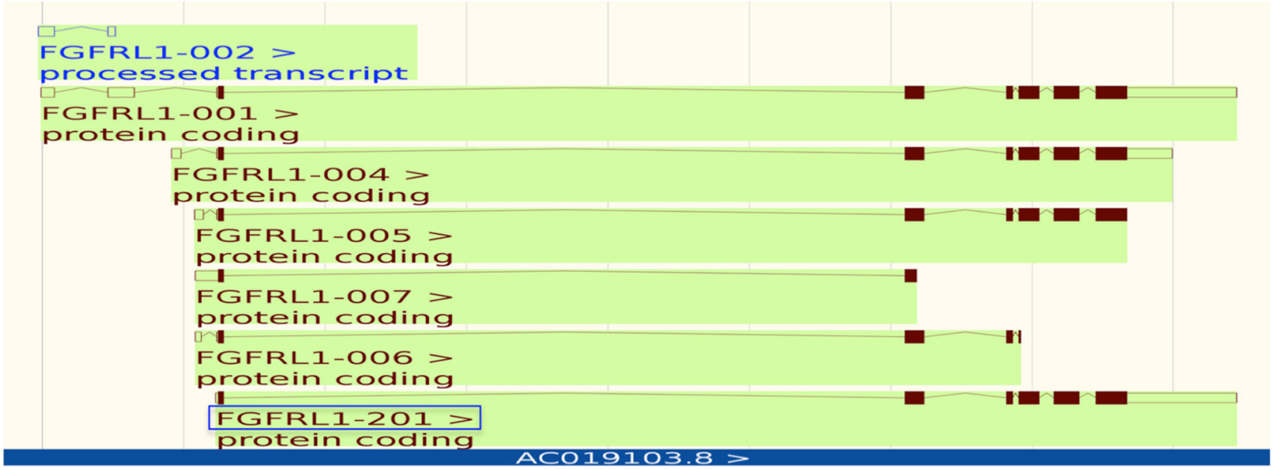

Gene name	ENST	TSS_ID	ISOFORM	CONTROL	PININ_KD	CTBP1_KD	CTBP2_KD
FGFRL1	ENST00000398484	TSS88468	FGFRL1-001	0.312617	0.0477062	0.0442208	0.041046
FGFRL1	ENST00000512562	TSS88468	FGFRL1-002	2.1013	0.0571443	0.771089	6.76E-06
FGFRL1	ENST00000510644	TSS88469	FGFRL1-004	2.74E-12	2.39E-44	1.63E-05	0.00030825 8
FGFRL1	ENST00000504138	TSS88470	FGFRL1-005	8.10E-89	2.60E-71	1.36E-73	3.39E-73
FGFRL1	ENST00000507339	TSS88470	FGFRL1-006	1.25E-244	2.04E-113	9.10E-195	3.42E-191
FGFRL1	ENST00000512174	TSS88470	FGFRL1-007	0	2.75E-175	0.0721087	0
FGFRL1	ENST00000264748	TSS88471	FGFRL1-201	41.8393	12.912	19.3411	11.7837
FGFRL1	ENSG00000127418	TSS88468	Unknown	0.0473909	0.0229453	1.75E-08	0.00893484
			Total FPKM:	4.43E+01	1.30E+01	2.02E+01	1.18E+01

Supplementary Figure S4: Isoform analysis shows that Pinin_KD, CtBP1_KD and CtBP2_KD cells had significant reduction of the major FGFRL1-coding isoform, which contributed to the reduced expression of the gene. The exon intron structures of all the isoforms are shown in the top panel. Protein coding isoforms are shown in brown, whereas noncoding isoforms are shown in blue. The isoform with reduced expression in the knockdown cells is bracketed. The FPKM counts of the isoforms in different cell lines are shown in the bottom panel. The major protein coding isoform is shown in yellow, and the isoform with significant reduction in FPKMs in the knockdown cell lines are shown in blue.

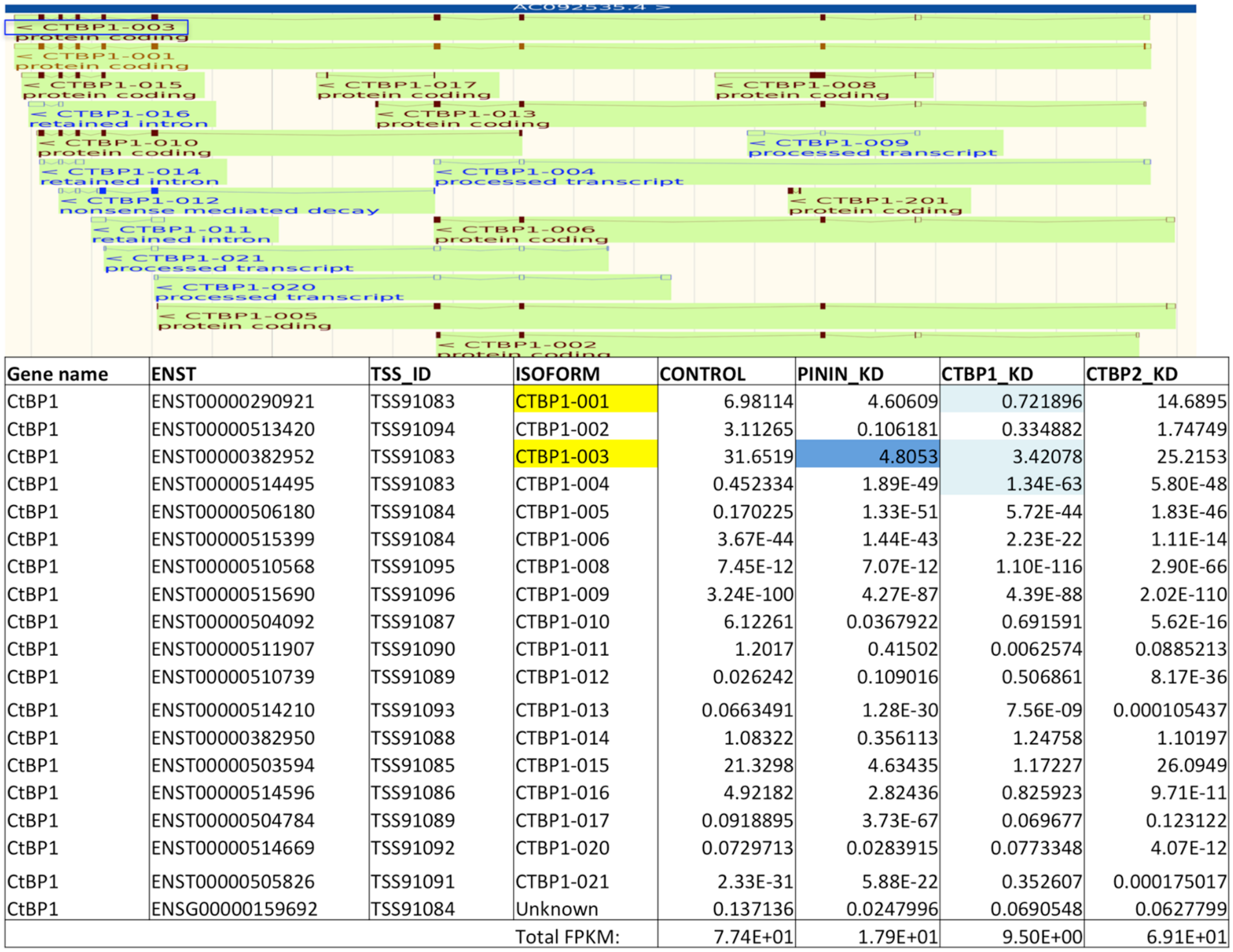

Gene name	ENST	TSS_ID	ISOFORM	CONTROL	PININ_KD	CTBP1_KD	CTBP2_KD
CtBP1	ENST00000290921	TSS91083	CTBP1-001	6.98114	4.60609	0.721896	14.6895
CtBP1	ENST00000513420	TSS91094	CTBP1-002	3.11265	0.106181	0.334882	1.74749
CtBP1	ENST00000382952	TSS91083	CTBP1-003	31.6519	4.8053	3.42078	25.2153
CtBP1	ENST00000514495	TSS91083	CTBP1-004	0.452334	1.89E-49	1.34E-63	5.80E-48
CtBP1	ENST00000506180	TSS91084	CTBP1-005	0.170225	1.33E-51	5.72E-44	1.83E-46
CtBP1	ENST00000515399	TSS91084	CTBP1-006	3.67E-44	1.44E-43	2.23E-22	1.11E-14
CtBP1	ENST00000510568	TSS91095	CTBP1-008	7.45E-12	7.07E-12	1.10E-116	2.90E-66
CtBP1	ENST00000515690	TSS91096	CTBP1-009	3.24E-100	4.27E-87	4.39E-88	2.02E-110
CtBP1	ENST00000504092	TSS91087	CTBP1-010	6.12261	0.0367922	0.691591	5.62E-16
CtBP1	ENST00000511907	TSS91090	CTBP1-011	1.2017	0.41502	0.0062574	0.0885213
CtBP1	ENST00000510739	TSS91089	CTBP1-012	0.026242	0.109016	0.506861	8.17E-36
CtBP1	ENST00000514210	TSS91093	CTBP1-013	0.0663491	1.28E-30	7.56E-09	0.000105437
CtBP1	ENST00000382950	TSS91088	CTBP1-014	1.08322	0.356113	1.24758	1.10197
CtBP1	ENST00000503594	TSS91085	CTBP1-015	21.3298	4.63435	1.17227	26.0949
CtBP1	ENST00000514596	TSS91086	CTBP1-016	4.92182	2.82436	0.825923	9.71E-11
CtBP1	ENST00000504784	TSS91089	CTBP1-017	0.0918895	3.73E-67	0.069677	0.123122
CtBP1	ENST00000514669	TSS91092	CTBP1-020	0.0729713	0.0283915	0.0773348	4.07E-12
CtBP1	ENST00000505826	TSS91091	CTBP1-021	2.33E-31	5.88E-22	0.352607	0.000175017
CtBP1	ENSG00000159692	TSS91084	Unknown	0.137136	0.0247996	0.0690548	0.0627799
			Total FPKM:	7.74E+01	1.79E+01	9.50E+00	6.91E+01

Supplementary Figure S5: Isoform analysis of the CtBP1 transcripts shows that Pinin_KD cancer cells had significant reduction of one major CtBP1-coding isoform, which contributed to the reduced expression of the gene. The legend for this figure is the same as for Supplementary Figure S4. For the CtBP1_KD cell line, though not significant at the individual level, many of the major coding isoforms were reduced (shaded in light blue), suggesting the effect of shRNA on reduced expression of CtBP1 in this cell line.

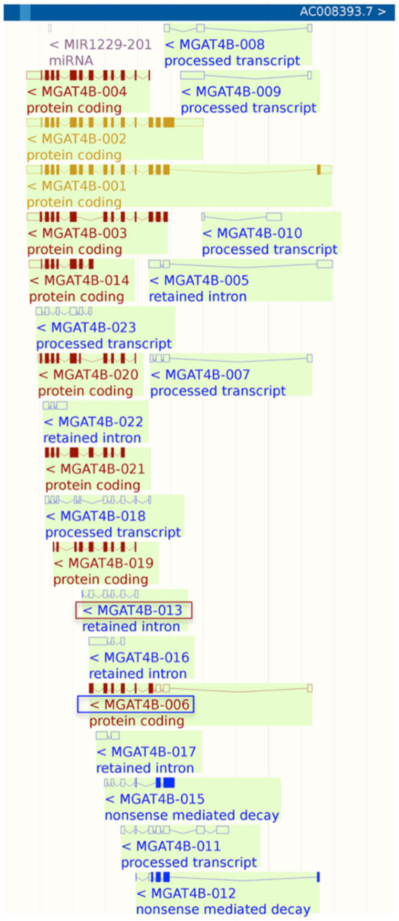

Gene name	ENST	TSS_ID	ISOFORM	CONTROL	PININ_KD	CTBP1_KD	CTBP2_KD
MGAT4B	ENST00000518778	TSS99514	MGAT4B-004	36.9992	85.6434	42.7703	30.0071
MGAT4B	ENST00000337755	TSS99515	MGAT4B-002	2.36703	0.991999	1.16635	1.26516
MGAT4B	ENST00000292591	TSS99516	MGAT4B-001	60.5014	32.0125	59.7096	36.762
MGAT4B	ENSG00000161013	TSS99516	Unknown	0.645841	0.581636	0.812026	0.414383
MGAT4B	ENST00000519836	TSS99517	MGAT4B-003	11.4546	31.1992	19.6672	10.8597
MGAT4B	ENST00000520969	TSS99518	MGAT4B-014	35.7007	80.3232	57.6945	28.0776
MGAT4B	ENST00000522293	TSS99518	MGAT4B-023	2.19E-11	0.0745996	1.22668	2.23E-10
MGAT4B	ENST00000518980	TSS99519	MGAT4B-020	2.46506	0.494215	7.49852	3.66309
MGAT4B	ENST00000522451	TSS99520	MGAT4B-022	0.448716	0.778727	0.456415	0.647543
MGAT4B	ENST00000520875	TSS99521	MGAT4B-021	1.2562	0.900088	2.70367	0.81088
MGAT4B	ENST00000523382	TSS99514	MGAT4B-018	3.49531	2.45331	3.12837	0.337453
MGAT4B	ENST00000518867	TSS99519	MGAT4B-019	0.218182	1.03831	2.19478	0.85582
MGAT4B	ENST00000518702	TSS99519	MGAT4B-013	2.95E-27	1.99E-52	1.43631	6.47E-31
MGAT4B	ENST00000520822	TSS99521	MGAT4B-016	1.97643	1.47213	2.57039	0.970055
MGAT4B	ENST00000523108	TSS99522	MGAT4B-006	0.921896	3.99E-51	1.58879	1.09E-27
MGAT4B	ENST00000521855	TSS99523	MGAT4B-017	1.51316	0.248636	0.546915	0.531197
MGAT4B	ENST00000520918	TSS99524	MGAT4B-015	1.95883	6.26E-27	0.308341	1.13E-23
MGAT4B	ENST00000521305	TSS99525	MGAT4B-011	8.37E-28	6.50E-05	9.72E-102	0.224628
MGAT4B	ENST00000523329	TSS99526	MGAT4B-012	3.91E-97	0.0450588	1.55E-82	0.459497
MGAT4B	ENST00000518168	TSS99516	MGAT4B-005	9.77E-87	0.305016	5.26E-56	0.211133
MGAT4B	ENST00000520134	TSS99522	MGAT4B-007	0.415941	5.73E-44	0.112954	9.89E-272
MGAT4B	ENST00000519616	TSS99522	MGAT4B-008	0.40766	0.065816	0.112004	0.08733
MGAT4B	ENST00000520019	TSS99522	MGAT4B-009	1.97E-18	0.0134416	0.000000126	3.70E-53
MGAT4B	ENST00000519965	TSS99527	MGAT4B-010	8.31E-32	2.59E-56	0.489332	0.790856
			Total FPKM:	1.63E+02	2.39E+02	2.06E+02	1.17E+02

Supplementary Figure S6: Isoform analysis of the MGAT4B transcripts shows that the isoforms with significantly altered expression in the knockdown cells are not the major coding isoforms and may not affect the expression of the gene. The legend for this figure is the same as for Supplementary Figure S4. The upregulated isoform and the downregulated isoform are bracketed or shaded in pink and blue, respectively.

Supplementary Table S1: Significant genes that are differentially expressed between Control and Pinin_KD, CtBP1_KD, and CtBP2_KD cancer cells.

See Supplementary File 1

Supplementary Table S2: Significant transcripts that are differentially expressed between Control and Pinin_KD, CtBP1_KD, and CtBP2_KD cancer cells.

See Supplementary File 2
